# Cost Burden of Post Stroke Condition in Nigeria: A Pilot Study

**DOI:** 10.5539/gjhs.v4n6p17

**Published:** 2012-08-15

**Authors:** Bridget N Birabi, Kayode Israel Oke, Paul O. Dienye, Udoka Chris Okafor

**Affiliations:** 1 Department of Medical Rehabilitation, College of Medical Sciences, University of Maiduguri, Maiduguri, Nigeria; 2 Department of Physiotherapy, University of Benin Teaching Hospital, Benin City, Nigeria; 3 Department of Family Medicine, University of PortHarcourt Teaching Hospital, PortHarcourt, Nigeria; 4 Department of Physiotherapy, College of Medicine, University of Lagos, Lagos, Nigeria

**Keywords:** stroke, cost burden, Nigeria

## Abstract

**Aim:**

Estimation of cost burden of a disease condition is a very important part of health care policy making worldwide. Till now, such documents are lacking especially on non-communicable diseases in the health policy making process in Nigeria. This article therefore attempts to report the results of a prospective cross-sectional study on the cost burden of a cerebrovascular accident condition (stroke) in Nigeria. It estimates the direct health care cost for a minimum period of 12weeks and maximum of 36weeks for post stroke hemiplegia.

**Study Design/Setting:**

It was a collaborative cross-sectional study amongst centers situated in urban and sub-urban environments in Southern Nigeria. It involved a hospital of an Oil and Gas Company in Port Harcourt, Nigeria, two Government tertiary hospitals in Port Harcourt and Benin-City, all in South-South Nigeria, the industrial hub of the country. A Private Specialist hospital in Lagos, South-West Nigeria, the corporate hub of the country was also included.

**Method:**

Patients diagnosed and admitted for management for cerebrovascular accident (stroke) in the above named health facilities formed the subjects of this study. Medical records (case files) of two hundred and forty (240) stroke patients managed within the last six years (2005- 2011) were randomly selected from the medical record departments of the study centers. Files of the patients who were admitted during acute care period (without discharge against medical advice) and were followed on out-patient basis without default within the study period were purposively utilized. The files were then assessed for the various investigations and treatment interventions of acute and long term care and the costs thereof. Ethical approval to access patients’ case files was sought and granted by the Research Ethics Committee of the different study centers.

**Results:**

The results revealed that it requires an average of N95,100: 00 ($600) and N767,900: 00 ($4860)in a government and a private hospital, respectively to access care within the first 36weeks of post stroke affectation in Nigeria.

**Conclusion:**

The outcome of this study suggests that managing stroke constitutes a huge direct cost burden unaffordable by an average Nigerian stroke sufferer. The implication is that lack of means for rehabilitative care may result in disability adjusted life years which further compounds burdens in terms of indirect cost on the sufferers’ and care givers’ productivity. It is therefore recommended that awareness of this disorder is created by policy makers and implementers where it does not exist and increased where it does with health promotion and preventive measures.

## 1. Introduction

Stroke is a global epidemic, and by no way a problem limited to western or high-income countries (Lopez, Mathers, Ezzati, Jamison, & Murray, 2006). It is the leading cause of morbidity and mortality in developed countries like the United States, imposing enormous economic burden on individuals and society overall (Taylor et al., 1996). Its emergence in developing countries like Nigeria creates a burden for concern. Stroke is a costly disease from human, family and societal perspectives. Starting from human costs, it is a leading cause of death and disability. Annually, about 16million first-ever strokes occur in the world, causing a total of 5.7 million deaths (Strong, Mathers, & Bonita, 2007). As a consequence, stroke ranks as the second cause of death in the world population after ischaemic heart disease [the third only if neoplastic diseases are considered as a group] (Di Carlo, 2009).

About 85% of all stroke deaths are registered in low- and middle-income countries, which also account for 87% of total losses due to stroke in terms of disability-adjusted life years (DALYs) calculated worldwide, in 72 million per year (Lopez et al., 2006). Stroke is a leading cause of long-term disabilities, ranging from motor control and urinary incontinence to depression and memory loss. The common ones being one sided paresis or paralysis and speech difficulties depending on which area of the brain the stroke has occurred. Stroke is also a leading cause of functional impairments, with 20% of survivors requiring institutional care after 3 months and 15%-30% being permanently disabled (Furie et al., 2011). Disuse atrophy of muscles and limitation of range of motion of joints of affected limbs result in chronic pain.

There are three major types of stroke: subarachnoid hemorrhage (SAH), intracerebral hemorrhage (ICH), and ischemic stroke [ISC] (Lopez et al., 2001). Present and future figures of stroke are strictly related to the demographic transition, occurring in both developed and developing countries. The world population aged 60 and over was 488 millions in 1990, and was projected to about 1,363 millions in 2030, with a percentage increase of 180% ^6^. In 1990, developing countries contained the 58% of the world elderly, while in 2030 about two-thirds of the total elderly population will be dwelling in these countries (World Bank Report, 2009).

The economic burden of stroke can be defined in terms of the direct costs of providing medical care to patients and the indirect costs associated with lost productivity. Studies of the economic burden of stroke typically use a prevalence-based approach to estimating the cost of stroke in a given year and often focus solely on the direct costs of care. Prevalence-based studies have been used to estimate the cost of treating stroke in Canada, Sweden, the United Kingdom, and the United States (Lopez et al., 2001). The most recent prevalence-based study of the cost of stroke in the United States, conducted by the Stroke Prevention Patient Outcomes Research Team, estimated the economic burden of stroke to be $30 billion in 1993, involving $17 billion in direct medical costs and $13 billion in indirect costs associated with lost earnings (Lopez et al., 2001).

Available facts and figures may easily explain why the economic burden of stroke is requiring increasing attention for more effective health care planning, implementation and resources allocation. An international comparison of stroke cost studies showed that, on average, 0.27% of gross domestic product was spent on stroke by national health systems, and stroke care accounted for ~3% of total health care expenditures (Strong, Mathers, & Bonita, 2007; Evers et al., 2004).

In the United States, the total direct and indirect costs of stroke for 2008 were estimated at $65.5 billion. Direct costs, which include the cost of physicians and other health professionals, acute and long-term care, medications and other medical durables, account for 67% of total costs. The remaining 33% is due to indirect costs, which consider lost productivity resulting from morbidity and mortality (Rosamond et al., 2008). In 27 EU countries, total annual cost of stroke is estimated at €27 billion: €18.5 billion (68.5%) for direct and €8.5 billion (31.5%) for indirect costs. A further sum of €11.1 billion is calculated for the value of informal care (European Cardiovascular Disease Statistics, 2008). Including informal care in the total amount, percentages would change to 48.6% for direct, 22.3% for indirect and 29.1% for informal care costs (Strong, Mathers & Bonita, 2007).

The cost of illness is defined as the personal cost of acute or chronic disease. The cost to the patient may be an economic, social or psychological cost or loss to himself, his family or community (www.mondofacto.com). The cost of illness may be reflected in absenteeism, productivity, response to treatment, peace of mind, quality of life, among others. It differs from health care cost which is restricted to the cost of providing services related to the delivery of health care rather than on impact of the personal life of the patient (www.mondofacto.com).

Cost of illness includes both direct and indirect costs (CDCP, 2004). Direct costs are medical and non-medical (physician visits, inpatients hospital stays, assistive devices, home modification and automobile modifications). Indirect costs could be calculated by estimating the value of productivity losses in workplaces and households that occur when persons with developmental disabilities die prematurely, are unable to work, or are limited in the amount or type of work they can perform (CDCP, 2004). Direct costs can also be actual currency expenditures by state and local governments, private individuals, families and groups and, philanthropic organizations towards current and future care as well as, prevention and treatment of an illness, disease or condition. Indirect costs reflect lost productivity of wage earners and sometimes homemakers as well as loss of productivity due to premature death (Lee, Chen, Yang, Liao, Lee et al., 2008).

Cost-of-illness studies measure the economic burden of a disease or diseases and estimate the maximum amount that could potentially be saved or gained if a disease were to be eradicated. Numerous cost-of-illness studies have been conducted over the past 30 years (Suaya, Shepard & Beatty, 2007; Leal, Luengo-Fernández, Gray, Peterson, & Rayner, 2006).

Many of these studies have been instrumental in public health policy debates because they highlight the magnitude of the impact of an illness on society or a part of society. Knowledge of the costs of an illness can help policy makers to decide which diseases need to be addressed first by health care and prevention policies. Additionally, these studies can indicate for which diseases cures would be valuable in reducing the burden of disease. For specific stakeholders, such as the federal government, cost-of-illness studies can show the financial impact a disease has on public programs, such as Medical Insurance. For employers, they can show which diseases have an especially large effect on their costs. Moreover, cost-of-illness studies provide important information for cost-effectiveness and cost-benefit analyses. Although only one part of cost analysis, cost-of-illness studies can provide a framework for the cost estimation in this analysis, the value of cost-of-illness studies can be seen in their frequent use by policy makers in the western economies.

However, despite the clinical and economic importance of post-stroke hemiplegia, to the best of the researchers’ knowledge, there is dearth of documentation on cost burden of post stroke hemiplegia in Nigeria as evidenced by the futility in the database searches of Medline, PubMed, Science Direct and SA ePublications. This disorder is disabling, usually requiring long term care and the cost of care is enormous. This pilot study is therefore a starting point aimed at addressing this existing gap in knowledge and research effort in Nigeria.

## 2. Method

### 2.1 Study Design/Settings

The present study was a retrospective study collaborating data from centers situated within urban and sub-urban environments of Nigeria. The collaboration centers included an Oil and Gas Company Hospital based in Port Harcourt, Nigeria, two federal government tertiary hospitals in Port Harcourt and Benin-City, all in South-South Nigeria and a private specialist hospital in Lagos, South-West Nigeria. Ethical approval to access patients’ case files was sought and granted by the Research Ethics Committee of the different study centers.

### 2.2 Procedures

The files were then assessed for the various investigations and interventions and the costs thereof. The perspective of the study involved direct costs (micro-costing) of acute and long term care, procedures and investigations like the Brain Computed Tomographic (CT) Scan, Magnetic Resonance Imaging (MRI), Electrocardiogram (ECG), Chest and Skull x-rays, Carotid Ultrasound Doppler, Echocardiogram, Laboratory investigations, Drugs and Physiotherapy excluding assistive devices were obtained and recorded. Other costs borne on pain relief creams/gels which are patient specific in type and number used, Nursing, Bed fees and Feeding were also excluded. The study was delimited to case records of patients still in active employment (work), either institution or self employed, and who received care until formally discharged. Records of patients who had delayed attention/care due to financial difficulties, discharged against medical advice and those with paucity of details of management were also excluded from the study. All non-health service and informal costs were also excluded from the study.

### 2.3 Statistical Analysis

Data obtained were fed into SPSS version 16 and descriptive statistics of mean, standard deviation and percentage were used for analysis.

## 3. Results

A total of twenty-nine (29) patients’ case files were studied. They consisted of nineteen (19) (65.5%) males and ten (10) (34.5%) females whose ages ranged between 34yrs – 62yrs (mean= 49.66 ± 7.46yrs). The patients were managed for minimum of 12 weeks and maximum of 36 weeks post affectation. The result of the study revealed that immediate post-stroke hemiplegia medical investigations cost between N58,400: 00 ($370= €279.16) and N196,000: 00 ($1240= €935.57) for investigations that include MRI, laboratory investigations and ECG for an average patient. It also revealed that an average patient spent between N32,000: 00 ($202= e€152.41) and N200,000: 00 ($1265= €954.43) for physiotherapy intervention within 12weeks, and between N89,000:00 ($560= €422.51) and N560,000: 00 ($3540= €2670.89) for thirty-six weeks respectively post stroke affectation. A patient required between a sum of N4,700: 00 ($30= €22.63) and N11,900: 00 ($75= €22.63) for medications for a period between seven and fourteen days immediate post incidence. The minimum and the maximum amounts represent the average government and private health facilities costs respectively. Table 1 reflects these results accordingly. The factor of minimum and maximum was observed as a result of lower charges in government hospitals in comparison to company and private hospitals.

**Table 1:** Summary of key data of the results showing cost of investigations, drugs and physiotherapy

Variables	Mean	Range
**Age (years)**	48	34- 62
**Duration of treatment (months)**	24	12- 36
**Medical investigations (MRI, CT Scan, Laboratory, ECG, etc) cost**	N127,200: $805	N58,400: 00- N196,000: 00 $370- $1240
**Physiotherapy cost**	N220, 250: 00 $1391.75	$202- $3540
**Medication cost**	N8300 $52.5	$30- $75

N= Nigeria naira; $= America; MRI= Magnetic Resonance Imaging; CT= Computerized Tomography; ECG= Electrocardiography

## 4. Discussion

To the best of the authors’ knowledge, this study represents the first known attempt in Nigeria and possibly in West African at estimating the cost burden of a neuromuscular condition like stroke. To this end, there is paucity of local data to support the findings of this study. Indirect costs of productivity costs due to work loss and costs of care giving and travel time were not included in this analysis. All costs as used in this study are converted to and reported in 2012 US dollars equivalent to present its international value. To convert costs from the Nigeria naira to the US dollar, costs were converted using the mean international exchange rate for the current year in which each of the Nigeria costs was reported. The current prevalence of stroke in Nigeria is 1.14 per 1000 (Wahab, 2008) and reports show that stroke accounts for 0.9-4.0% of all admissions and 0.5-45% of all neurological admissions in Nigeria (Ogun, Ojini, Ogungbo, Kolapo & Danesi, 2005). This prevalence is similar to what is obtainable in a more populated country like India (Bharucha & Kuruvilla, 1998). There was a prevalence rate of 2.69 per 1000 persons in the US irrespective of race as at 2001 (Williams, 2001). Using these data for cost estimation, it is believed that an average of $62, 217 is required as direct medical cost for managing a stroke patient in a year excluding the cost of nursing care. The mean lifetime cost resulting from an ischemic stroke is estimated at $140,000 per patient in the United States in 2005 while it is estimated that costs related to stroke was expected to reach $62.7 billion Nationwide in the US in 2007 (Rosamond, Flegal, Friday et al., 2007). Stroke costs the Canadian economy $3.6 billion a year in physician services, hospital costs, lost wages, and decreased productivity (2000 statistic) (THDSC, 2009). A model estimate of £15 306 was made as cost (2001/2002 prices) to the NHS in the UK for every patient who experiences a stroke for over 5 years for acute care costs and institutional and home care costs (Youman, Wilson, Harraf & Kalra, 2003). Report indicated that the overall cost per person with a disorder of the brain like stroke in the US in 2010 was $3538 Gustavsson, Svensson, Jacobi, Allgulander, Alonso et al., 2011). Putting Nigeria population at 157million people and using the prevalence rate of 1.14 per 1000, it is estimated that an average of $1.1 billion (N173.8billion) is required as direct cost-of-illness excluding nursing care for stroke victims per annum in Nigeria. This is a huge amount for a country with poor economy which had a total health sector budget of $1.78billion for the year 2011. The cost of management analysis in this study revealed that forty-six percent 46% ($734) of the total direct cost of managing stroke in the acute and immediate post acute stages excluding nursing care charges is spent on physiotherapy (rehabilitation) services alone. The amount is huge for an average citizen who lives on per capital income of less than $2 per day and may make follow up on management a mirage which will subsequently result in disability-adjusted life year (DALY) in an average patient.

## 5. Conclusion and Recommendations

In conclusion, the authors believe that the findings in this study has established a template on which further studies in this area can be built to ensure efficient use of resources devoted to cost-effective treatments. It has also stimulated further exploration or thinking on the need to develop data that will assist government, non-governmental organizations with interest in preventive and rehabilitation medicine, healthcare providers and careers in planning, budgeting and implementation of healthcare programmes. It is recommended that the government should put in place strengthened medical insurance programme that can assist stroke sufferers access medical care especially physiotherapy care at affordable or highly subsidized rates that can enable sufferers recover from this condition without disabilities. Increased funding of acute stroke medical care and rehabilitation services in hospitals is also advocated. There is also a need to strengthen health education programs to inform citizens on how to engage in adequate and structured physical activity measures to prevent the occurrence of this condition. Further studies that would explore both direct and indirect cost of care of post stroke condition both at the acute and long-term stages in Nigeria are also recommended.
